# Supplementary material for: Desiccation-tolerant Acinetobacter as a robust chassis candidate for gas-phase bioprocesses
Source: J Biol Eng. 2026 Mar 23;20:80. doi: 10.1186/s13036-026-00668-3 (PMC13130816; doi:10.1186/s13036-026-00668-3)
Supplement: Supplementary file 1 — Supplementary Material 1 [file 13036_2026_668_MOESM1_ESM.pdf]

Supplementary Information for

Desiccation-tolerant *Acinetobacter* as a robust chassis candidate for gas-phase bioprocesses

Shogo Yoshimoto, Hayata Yamada, Shori Inoue, Katsutoshi Hori*

Department of Biomolecular Engineering, Graduate School of Engineering, Nagoya University, Furo-cho, Chikusa-ku, Nagoya, Aichi 464-8603, Japan

* Correspondence: Katsutoshi Hori

Department of Biomolecular Engineering, Graduate School of Engineering, Nagoya University, Furo-cho, Chikusa-ku, Nagoya, Aichi 464-8603, Japan.

Tel: +81-52-789-3339

Email: khori@chembio.nagoya-u.ac.jp

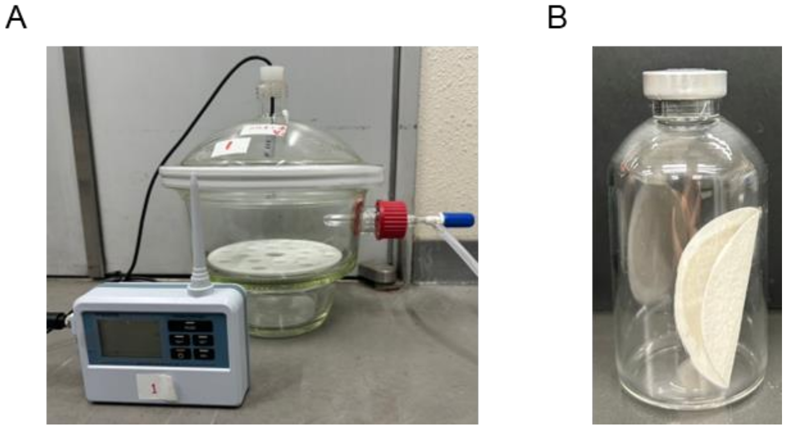

Figure S1. Photographs of the experimental setup. (A) A desiccator connected to a humidity data logger used for controlled cell desiccation. (B) A vial containing a glass filter with immobilized cells for the toluene degradation assay.

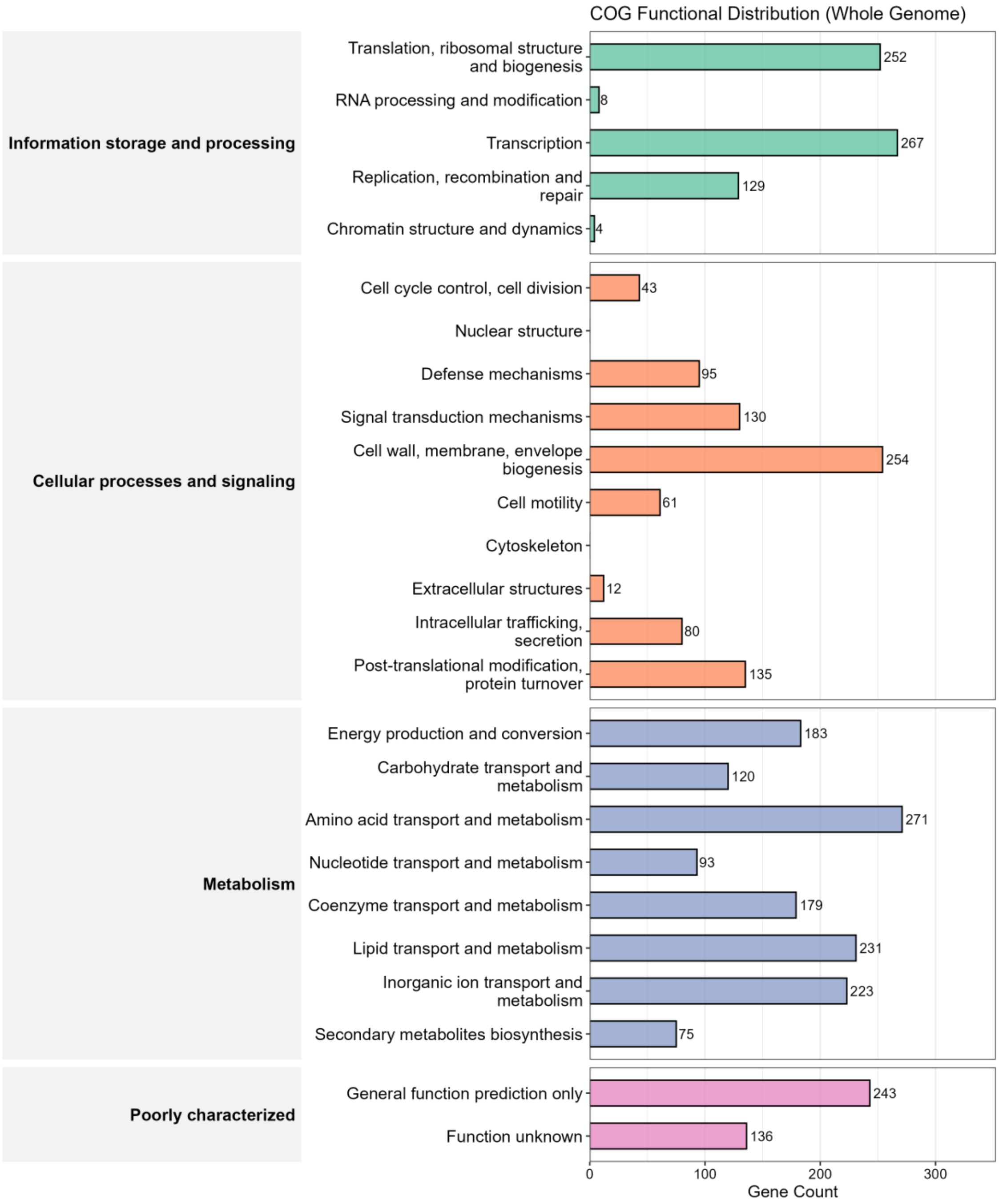

Figure S2. COG functional classification of the complete genome of Tol 5. All predicted protein-coding sequences (CDSs) in the Tol 5 genome were assigned to COG functional categories. The bar chart represents the number of genes assigned to each functional category.

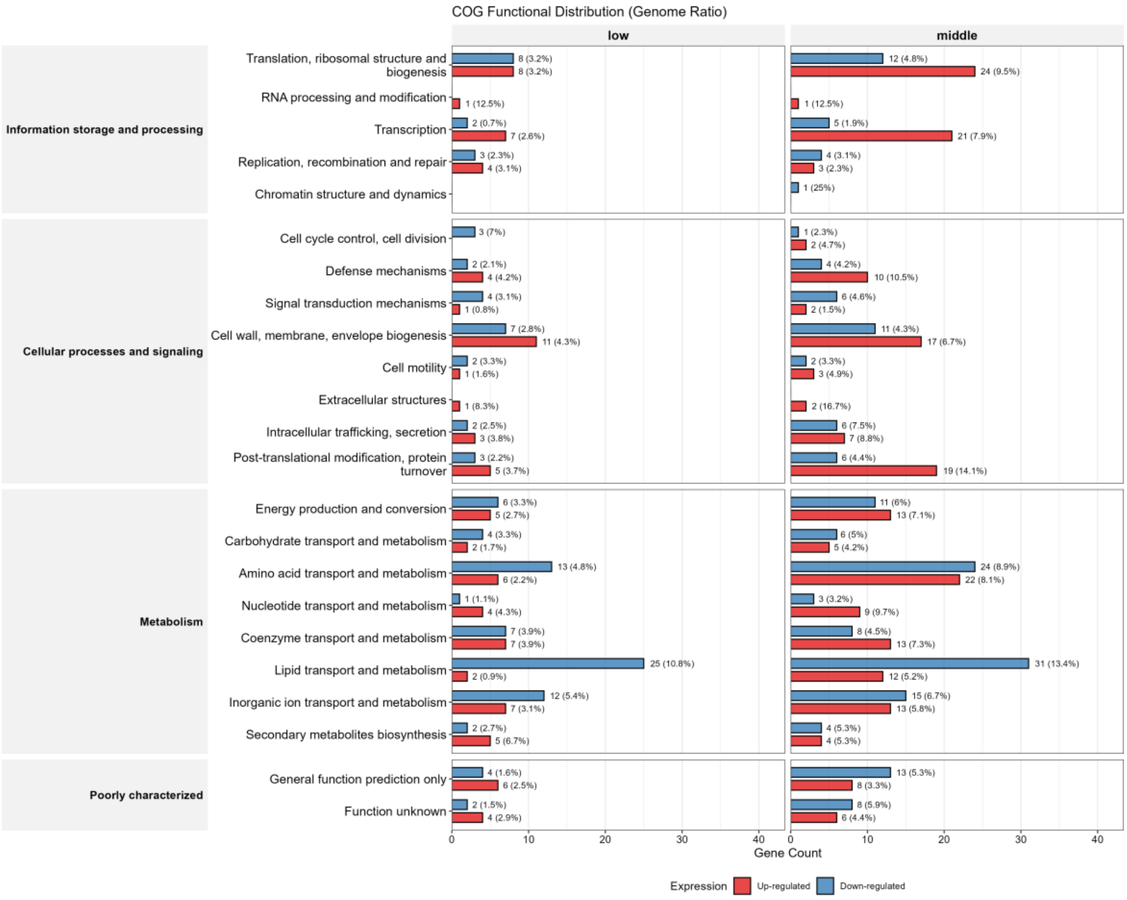

Figure S3. COG functional distribution of differentially expressed genes (DEGs) identified by RNA-Seq. DEGs identified under low (8% RH) or middle (52% RH) humidity conditions relative to high (>95% RH) humidity condition were assigned to COG functional categories. Red and blue bars represent the number of upregulated and downregulated genes, respectively. The labels next to each bar indicate the absolute gene count, followed by the percentage (in parentheses) of these DEGs relative to the total number of genes assigned to that category in the genome of Tol 5.

Table S1. Statistical analysis for the CFU counting.

Table S2. Statistical analysis for the ATP assay.

Table S3. Gene expression in Tol 5 under different desiccation conditions.

Table S4. DEGs assigned to the “Information storage and processing” supercategory.

Table S5. DEGs assigned to the “Cellular processes and signaling” supercategory.

Table S6. DEGs assigned to the “Metabolism” supercategory.

Table S7. DEGs classified as “Poorly characterized.”

Table S8. DEGs unassigned to any COG category.

Table S9. Genes assigned to the “Energy production and conversion” category.
